# How prepared are dental students to manage medical emergencies, a cross-sectional survey from Saudi Arabia

**DOI:** 10.3389/fmed.2025.1715028

**Published:** 2026-01-07

**Authors:** Albraa B. Alolayan, Ahmad A. Othman, Asma A. Bakkari, Arwa M. Hafiz, Aljawharah M. Alanazi, Amaal M. Alroithi, Rahaf H. Aljohani, Najla Dar-Odeh

**Affiliations:** 1 Department of Oral and Maxillofacial Diagnostic Sciences, College of Dentistry, Taibah University, Madinah, Saudi Arabia; 2 Saudi board Orthodontics and Dentofacial Orthopedics Resident, King Abdullah Bin Abdulaziz Hospital, Riyadh, Saudi Arabia; 3 Saudi Board Pediatric Dentistry Resident, King Fahad Hospital, Madinah Health Cluster, Madinah, Saudi Arabia; 4 Department of Orthodontics, Faculty of Dentistry, King Abdulaziz University, Jeddah, Saudi Arabia; 5 Saudi Board Oral and Maxillofacial Surgery Resident, Ministry of National Guard Health Affairs, King Abdul Aziz Medical City, Jeddah, Saudi Arabia; 6 Saudi Board Pediatric Dentistry Resident, Batterjee Medical College, Jeddah, Saudi Arabia; 7 School of Dentistry, The University of Jordan, Amman, Jordan

**Keywords:** medical emergencies, dental students, knowledge, attitude, confidence, dental internships, Saudi Arabia

## Abstract

**Background:**

Medical emergencies (MEs) in dental practice, though rare, can occur posing significant risks to patients. Dental professionals should be well-prepared to manage such situations. This study aimed to assess the knowledge, self-perceived confidence and preparedness of dental students and interns regarding the management of MEs in dental settings.

**Methods:**

A questionnaire-based cross-sectional study was conducted among clinical dental students and interns at a dental college in Saudi Arabia. Data were collected using an anonymous, self-administered online questionnaire consisting of 20 multiple-choice questions assessing knowledge, preparedness and confidence in handling MEs. Statistical analysis was performed using SPSS and employing Pearson’s Chi-squared test and logistic regression analysis.

**Results:**

The study analyzed data from 145 out of 200 participants invited (response rate = 72.5%). Most participants were female (51.0%) and aged ≥ 24 years (62.8%). A high knowledge level was identified in 58.6% of participating students and interns. While females exhibited a slightly higher knowledge than males, higher confidence and preparedness levels were identified among males, though the difference was not statistically significant (*p* = 0.05). Older and more advanced students demonstrated significantly higher knowledge and confidence (*p* < 0.05). The most encountered emergency by participants during dental treatment was syncope (65.5%), and seizures (29.6%). Adrenaline was the most commonly cited emergency drug in terms of availability in dental practices (38.6%), followed by anti-histamines (23.4%), while atropine was the least available (13.8%). Notably, 58.6% of participants reported lack of these emergency drugs in their practice settings.

**Conclusion:**

The study highlighted a gap in the preparedness of dental students and interns to manage MEs effectively. The findings emphasize the need for enhanced training and education, particularly in managing more severe medical emergencies, to improve the overall emergency response in dental practices. Further, efforts should focus on increasing access to emergency drugs and equipment, along with regular BLS training, to ensure optimal patient safety.

## 1 Introduction

The field of dentistry continues to evolve, both in terms of procedures and techniques, as well as the diverse medical background of the dental patient population. Thanks to advancements in medical care, individuals are living longer and receiving treatments for conditions that were once considered fatal. As a result, the patient population seen in dental practices has become older, and affected by a wide variety of co-morbidities, making it essential for dental professionals to stay updated with the latest medical knowledge and emergency protocols (1–3).

Medical emergencies (MEs) in the dental setting are relatively rare, however, they can still occur. A medical emergency in a dental practice is typically the result of a sudden deterioration of a pre-existing medical condition. Such emergencies can present an immediate threat to a patient’s life, requiring prompt and decisive intervention (4). Further, a large proportion of dental patients are medically compromised, affected by one or more comorbidities such as cardiovascular or endocrine disorders (5). Given this, prevention is always the preferred approach to avoid such emergencies. The best way to prevent MEs in the dental clinic is through comprehensive pre-treatment planning, including taking an in-depth medical and drug history from patients. This allows the dentist to create a tailored treatment plan and make necessary adjustments to minimize the risk of medical complications during treatment (6).

Oral and maxillofacial surgeons often serve at the frontline of medical emergency management (7). Their ability to promptly recognize and initiate appropriate care is crucial, as they are frequently the sole clinicians available on site. Moreover, surgical interventions such as dental extraction represent the most common category of procedures associated with the occurrence of medical emergencies (8).

Research conducted by Azad et al. (9) highlights that the most common medical emergencies encountered before, during, and immediately after dental procedures include hyperventilation, convulsions, and hypoglycemia. These are followed by vasovagal syncope, angina pectoris, hypotension, hypersensitivity reactions, and adverse drug reactions. Understanding and preparing for these scenarios is crucial for dentists, as timely management can prevent serious complications.

Medical emergencies in dental practice are often precipitated by local anesthesia, which is the most common medication used by the dental community (10). Stressful procedures combined by patient anxiety can exacerbate medical conditions or trigger adverse reactions (11). This underscores the responsibility of dental professionals to receive training in recognizing and appropriately managing medical emergencies, including the acquisition of essential skills such as Basic Life Support (BLS), to ensure effective emergency management (12–14).

Establishing the knowledge base and developing necessary skills for management of medical emergencies should start as early as undergraduate dental years. Therefore, the present study aimed to assess the knowledge of clinical dental students and interns regarding management protocols for common medical emergencies. The study also aimed to evaluate their self-perceived confidence and preparedness in handling emergency situations.

## 2 Materials and methods

### 2.1 Study tool and participants

This study was a cross-sectional observational study conducted among dental students and interns in a public university western Saudi Arabia. Data were collected through an anonymous, self-administered online questionnaire. The questionnaire was designed by all co-authors using Google Forms, and a link was created. It was validated before commencing the study. A pilot study was carried out by distributing the questionnaire to 10 volunteer dental students at the same university on two occasions separated by 10 days to compare responses. Participants giving different answers to a specific question, were interviewed to revise questions and modify them where necessary to make them more understandable. The process was repeated until complete agreement between responses of each volunteer was achieved.

The questionnaire was in English, and it consisted of 20 closed-ended, multiple-choice questions, and divided into three sections. First section was on demographics (age, gender, and study level) (Table 1); second section was on knowledge about managing specific emergency scenarios (syncope, airway obstruction, postoperative bleeding, anaphylaxis, epileptic seizures, angina, and CPR technique) (Table 2); and finally, a third section that explored past experience, practices, training needs, and confidence of students regarding medical emergencies (Tables 3, 4). The knowledge section consisted of nine questions, with each correct answer being assigned one point. A low level of knowledge was defined as correctly answering 1–4 questions, while a high level of knowledge was defined as correctly answering 5–9 questions. The practice level was assessed based on responses to six items. A low level was considered if the participant showed a correct response to 1–2 of the items, and a high level was considered if the participant showed a correct response to 3–6 items.

**TABLE 1:** Demographic data of participating clinical dental students and interns (*n* = 145).

Demographics	Frequency	Percent
**Gender**		
Female	74	51.0%
Male	71	49.0%
**Age**	(mean = 23.56, std. deviation = 1.433)	
< 24 years	54	37.2%
≥ 24 years	91	62.8%
**Study level**		
Dental intern	72	49.7%
4th year	36	24.8%
5th year	18	12.4%
6th year	19	13.1%

**TABLE 2:** Knowledge of dental students and interns regarding management of specific medical emergency scenarios that may be encountered in dental practice (*n* = 145).

Medical emergency scenario/information	Frequency	Percent
**A patient suffered from syncope when you commenced a dental procedure. What would be your immediate action?**		
Continue dental procedure	2	1.4%
Place the patient in Trendelenburg position and give ammonia inhalant*	89	61.4%
Make patient sit in an upright position	25	17.2%
None of the above	29	20.0%
**A patient is cited with airway obstruction during dental treatment due to aspiration of a foreign body. What would you do?**		
Attempt Heimlich maneuver	15	10.3%
Examine mouth and local area	6	4.1%
Ask the patient to cough	32	22.1%
All of the above*	92	63.4%
**You confirm that your patient remains unresponsive despite shaking and shouting. What will be your immediate action?**		
Start CPR	74	51.0%
Activate EMS*	41	28.3%
Put in recovery position	27	18.6%
Observe	3	2.1%
What is the location of chest compression?		
Left side of the chest	16	11.0%
Right side of the chest	8	5.5%
Mid chest*	88	60.7%
Xiphisternum	33	22.8%
**How many chest compressions and breaths should be given in CPR when performed by a single rescuer?**		
30 chest compressions and 2 rescue breaths at a rate of 100–200 compressions per minute*	88	60.7%
30 chest compressions and 1 rescue breath	32	22.1%
10 chest compressions and 1 rescue breath	18	12.4%
None of the above	7	4.8%
**In the event of spontaneous bleeding following extraction, what would be your primary management?**		
Ask the patient to bite firmly on the gauze for 30 min	99	68.3%
Ask the patient to bite firmly on a tea bag for 30 min	6	4.1%
Have cold drinks without using a straw because using a straw causes negative pressure	5	3.4%
All of the above*	35	24.1%
What is the first drug of choice in anaphylaxis?		
Corticosteroids	15	10.3%
Adrenaline	76	52.4%
Vasodilators	6	4.1%
Anti-histamine*	48	33.1%
**What will be your primary management in case of epileptic fits in the dental chair?**		
Continue dental procedure	–	–
Make the patient lie on the lateral position and wait for seizures to end*	114	78.6%
Inject IV diazepam	13	9.0%
None of the above	18	12.4%
**When a patient presents with anginal pain, which of the following drugs should be administered?**		
Adrenaline	8	5.5%
Sublingual nitrates*	104	71.7%
NSAIDs	16	11.0%
None of the above	17	11.7%

*Correct answers.

**TABLE 3:** Emergency situations encountered by participants, and emergency drugs availability in dental clinics as reported by students and interns (*n* = 145).

Emergency	Frequency	Percentage
Syncope	95	65.5%
Seizure	43	29.6%
Bronchospasm	6	4.1%
None of the above	4	2.7%
**Emergency drugs**	**Frequency**	**Percentage**
Adrenaline	56	38.6%
Anti-histamines	34	23.4%
Hydrocortisone	23	15.9%
Atropine	20	13.8%
None of the above	85	58.6%

**TABLE 4:** Preparedness and confidence of dental students and interns in managing medical emergencies (*n* = 145).

Question	Frequency	Percentage
**Have you attended workshops on Basic Life Support (BLS)?**		
Yes	142	97.9%
No	3	2.1%
**Do you think you are prepared for managing medical emergencies?**		
Yes	46	31.7%
May be	74	51.0%
No	25	17.2%
**Do you enquire about medical history including medication and allergy?**		
Yes	119	82.1%
Maybe	19	13.1%
No	7	4.8%
**Do you obtain vital signs (BP, pulse rate, respiration, and temperature) before commencing any treatment?**		
Yes	26	17.9%
Sometimes	86	59.3%
No	33	22.8%
**Do you think you can manage any emergency condition in your dental office very confidently?**		
Yes	15	10.3%
May be	72	49.7%
No	58	40.0%
**Are emergency kits available in your dental office?**		
Yes	16	11.0%
No	69	47.6%
I don’t know	60	41.4%

The study recruited clinical dental students (fourth to sixth study year), and dental interns. All students were invited to participate while on campus at the Faculty of Dentistry, Taibah University during the period October-December 2023. Student representatives from each year group distributed the questionnaire to their peers. The data were collected anonymously to maintain participant confidentiality and encourage transparent responses.

Inclusion criteria were dental students in their 4th, 5th, and 6th years, as well as dental interns affiliated to the participating dental school. Pre-clinical students (first- 3rd-year dental students) were excluded. Using Epi Info™ Epi Info epidemiological software (CDC, Centers for Disease Control, Atlanta, United States), sample size was calculated based on a total population of 200 dental students and interns who were enrolled in the college at the time of the survey. A sample size of 140 was determined to provide a 97% confidence level, a 5% confidence limit, at a 50% expected knowledge level.

### 2.2. Ethics

Ethical approval was obtained from the research Ethics Committee of Taibah University, College of Dentistry (Number TUCDREC/20102020/NDar-Odeh), and the study was conducted in complete accordance with the principles of the World Medical Association Declaration of Helsinki. Before recruitment, study objectives were explained to participants and informed consent was obtained. Participants were also informed of the voluntary and confidential nature of participation.

### 2.3 Statistical analysis

The Statistical Package for the Social Sciences (SPSS v.26, Armonk, NY: IBM Corp.) was used to analyze the data in this study, including the calculation of frequencies and percentages to describe the sample characteristics. Pearson’s Chi-squared test (χ^2^) was employed to compare the results between genders and between students and interns regarding their management of medical emergencies. Additionally, logistic regression analysis was conducted to examine the association between knowledge and practice levels and the characteristics of participants, with a significance level set at *p* ≤ 0.05.

## 3 Results

Out of the 200 clinical-year dental students and interns invited, 145 completed and returned the questionnaire, yielding a response rate of 72.5%. Mean age was 23.56 years (±1.433). Slightly more female students participated (51.0%), with the age group (≥24 years) and dental interns representing most participants (62.8, 49.7%, respectively), (Table 1).

Table 2 presents the participants’ responses to questions assessing the knowledge on management of specific medical emergency scenarios. Most students (n = 85, 58.6%) demonstrated a high level of knowledge (Table 2).

When participants were asked about the emergency situations they had encountered in the dental chair, the most common emergencies were syncope (*n* = 95, 65.5%) followed by seizures (*n* = 43, 29.6%) (Table 3). Table 3 also presents the emergency drugs that are available in the dental setting as perceived by participants. Adrenaline (*n* = 56, 38.6%) and antihistamine (*n* = 34, 23.4%) were the most frequently reported drugs by participants.

Table 4 presents perceptions of participants regarding their preparedness in handling medical emergencies, and their practices regarding prevention of a medical emergency. Only 2% of participants have not attended a BLS workshop, while 51.0% were not confident about their preparedness for handling a medical emergency (Table 4). While approximately 80% stated that they enquire about medical history prior to dental treatment, only 17.9% consistently assessed vital signs of patients prior to therapy (Table 4).

Table 5 presents the results of comparing participants’ gender (male vs. female) regarding their knowledge and preparedness levels. The findings show that females had a slightly higher level of knowledge (59.5%) compared to 57.7% of males, while males had a higher level of preparedness (46.5%) compared to 39.2% of females. However, there was no statistically significant association (p > 0.05) between gender and either knowledge or preparedness (Table 5).

**TABLE 5:** Gender analysis of students and interns in knowledge and preparedness in the management of medical emergency (*n* = 145).

Gender	Knowledge		Preparedness	
	Low (0–4)	High (5–9)	Low (0–2)	High (3–6)
Female	30	44	45	29
% Within gender	40.5%	59.5%	60.8%	39.2%
Male	30	41	38	33
% Within gender	42.3%	57.7%	53.5%	46.5%
*p*-value	0.834		0.375	

Table 6 presents the results of the logistic regression model for knowledge and preparedness as dependent variables, with independent variables including gender, age, and clinical year of dental students as predictors. A significant impact was found for age in the knowledge model, with a positive beta coefficient value of 0.467 (*p* = 0.034). However, no significant impact was found for the logistic regression model regarding preparedness (*p* > 0.05).

**TABLE 6:** Result of logistic regression model for knowledge and preparedness by participants characteristics (*n* = 145).

Title 1	B	S.E.	Wald	df	Sig.	Exp (B)
**Knowledge**						
Constant	−11.052	5.418	4.162	1	0.041	0.000
Gender (1)	0.334	0.366	0.833	1	0.361	1.397
Age	0.467	0.220	4.507	1	0.034*	1.595
Study level: fourth year	0.423	0.714	0.351	1	0.554	1.526
Study level: fifth year	1.017	0.716	2.020	1	0.155	2.765
Study level: sixth year	0.169	0.556	0.093	1	0.761	1.184
**Preparedness**						
Constant	−5.360	4.872	1.211	1	0.271	0.005
Gender (1)	−0.276	0.354	0.607	1	0.436	0.759
Age	0.212	0.198	1.152	1	0.283	1.236
Study level: fourth year	0.447	0.663	0.455	1	0.500	1.564
Study level: fifth year	0.183	0.629	0.084	1	0.771	1.201
Study level: sixth year	0.567	0.537	1.116	1	0.291	1.763

Reference for gender: Male. Reference for study level: dental intern. *Significant at the 0.05 level.

## 4 Discussion

Management of medical emergencies in dental practice is a critical aspect of clinical practice, yet it remains an area that requires ongoing attention in dental education (1, 2). Saudi Arabia is witnessing a rapid growth in dental education, whereby 25 dental schools were established and approximately 1,500 dental practitioners graduating each year. As the population of medically compromised patients is increasing and as the complexity of dental procedures is rapidly developing, it was important to assess the knowledge and preparedness of future dentists in handling such emergencies.

This study targeted dental students throughout their clinical training years. They start their training on the management of MEs during the fourth year as part of the oral surgery didactic and clinical courses. They continue a more advanced training during the sixth year through the comprehensive clinical dentistry course, which consists of a didactic component, and case-based learning seminars throughout the year. Further, students are assessed at the end of the year by case scenario exam to evaluate their competency in the management of medical emergencies. Therefore, it was necessary to assess students’ knowledge and perceptions throughout their clinical training journey that extends over a 4-year period.

The findings of this study revealed a significant disparity in the level of knowledge among dental students and interns concerning the management of medical emergencies. Approximately one in two participants demonstrated a high level of knowledge, while 41.4% exhibited a low knowledge level, suggesting a potential gap in emergency care education. Recent studies have similarly identified deficiencies in students’ knowledge and awareness regarding the management of medical emergencies (MEs) (15–17). The findings also reveal areas warranting improvement, as students demonstrated varying levels of knowledge in particular emergency scenarios. Approximately 6–7 in 10 students were able to identify the appropriate procedure of CPR and the recommended primary management of syncope, airway obstruction due to aspiration, epileptic shock and angina. On the other hand, most were not knowledgeable about the immediate management of unresponsive individuals, and post-extraction bleeding. Further, most did not identify the appropriate emergency drug for anaphylaxis. These findings highlight the critical need for continuous education and training to ensure comprehensive disaster preparedness, as emphasized in previous studies (18, 19), particularly in the aspect of emergency medication selection. Consistent with these findings, a previous cross-sectional study at the same dental school, conducted between few years previously, revealed that only 54% of participants were knowledgeable about the correct management of common, life-threatening conditions. The study also found that 50–74% of participants demonstrated moderate knowledge, and nearly all students, along with 90% of trainers, expressed a need for further training (20). However, a more recent study conducted at another dental school in Saudi Arabia, involving 214 dental and medical students in their clinical years, concluded that the students had a strong understanding of emergency care fundamentals, with 78.6% being familiar with BLS protocols (21). This may indicate that dental schools are already recognizing the importance of medical emergency education and integrating that in clinical dental education.

When participants were asked about the emergency situations they had encountered while treating patients in the dental chair, the most common emergency reported was syncope, followed by seizures, and bronchospasm, which was the least commonly cited. Additionally, 65.5% of the participants reported having encountered other types of medical emergencies. Consistent with these findings, the most common medical emergencies encountered in Polish dental offices were vasovagal syncope, orthostatic hypotension, and hyperventilation crises (15). Similarly, vasovagal syncope is the most commonly encountered medical emergency in dental practices (22, 23). In Brazil, the most common medical emergency in dental clinics was presyncope, followed by orthostatic hypotension (23). Overall, while the prevalence of medical emergencies varies across different countries, syncope remains the most frequently reported emergency. Additionally, less severe emergencies are generally more common than serious ones. This highlights the importance of implementation of stress reduction protocols and addressing patient’s concerns by preparing them appropriately to receiving various dental procedures. Regarding availability of emergency medications, this study identified adrenaline as the most frequently available emergency drug, followed by anti-histamines and hydrocortisone. In contrast, atropine was the least available drug, with only 17.2% of participants reporting its availability. Notably, approximately 60% of the total sample did not have any of these emergency drugs available in their practice settings. The lack of emergency equipment and drugs in many dental clinics worldwide may stem from factors such as insufficient experience with their use, underestimation of their importance, high costs, and the absence of regulations requiring lifesaving equipment in dental practices. A study in neighboring Jordan found that more than 75% of dentists had never used emergency medications in their clinics. Among those with emergency medications, adrenaline and steroids were the most commonly reported (6). Similar results can be seen in studies examining the use of equipment that assess vital signs. A study from the western region of Saudi Arabia found that only 22.9% of dental clinics had a blood pressure monitor (24), while in Lithuania, the blood pressure monitor was the most commonly available emergency equipment (25). In Nigeria, 91.1% of respondents reported not having an emergency kit in their clinics (26). These findings reveal a significant gap in emergency preparedness, with many dental clinics lacking essential medications and equipment needed to manage life-threatening situations

In this study most students showed a low level of confidence in handling various medical emergencies. A similar finding was reported by Al Ghanam et al. (6), where only one in two of participants expressed confidence in managing minor medical emergencies, while less than 10% felt fully capable of managing more serious emergencies. Comparable results have been observed in other studies, particularly in performing the important procedure of CPR. For example, an analysis of Brazilian dentists’ self-assessed abilities found that 79.7% did not feel capable of providing initial management for acute myocardial infarction, and 68.7% felt the same about cardiac arrest (23). In Kuwait, the majority of dentists had inadequate knowledge of CPR and only 57.2% felt competent in performing it (27). Further, in another study from Saudi Arabia less than 50% of dentists considered themselves skilled in performing CPR (28), and in Slovenia, only 51% of the participating dentists estimated that they were competent to provide CPR (29). These results show that continuous consistent training is mandatory to the dentist to stay self-confident and skillful in performing CPR and managing MEs. Other studies examining the self-confidence of dental practitioners in emergency management revealed that dentists are generally more confident in handling minor emergencies, probably due to their familiarity with conditions they encounter more frequently, as opposed to rare, severe emergencies (30, 31). These findings underscore the need for targeted interventions, such as additional training and simulation exercises, to boost the confidence and preparedness of those with lower levels of attitude and confidence, ensuring that all dental students are equally equipped to handle medical emergencies in clinical settings.

The study detected no significant difference in the level of knowledge and preparedness based on gender, although males showed a slightly higher level of self-confidence in managing medical emergencies. These findings align with results from other studies (9, 32). In contrast, age and study level were found to be predictors for the level of knowledge and preparedness among students. Specifically, age had a significant impact on knowledge, indicating that older students tended to have better knowledge in managing medical emergencies. This is consistent with the findings of Kharsan et al. (33) who concluded that awareness was greater among senior students and more experienced dentists. This indicates that factors such as age and experience, especially as students advance through their clinical years, may contribute to improved knowledge and confidence in managing medical emergencies.

One of the key strengths of this study is the comprehensive analysis provided by the logistic regression model, which allowed for the evaluation of the impact of multiple independent variables, such as gender, age, and study level, on dental students’ knowledge and perceptions toward medical emergency management. The study’s findings, particularly the significant impact of age on knowledge, provide valuable insights into how experience and maturity can enhance understanding of crucial concepts, such as the importance of obtaining a patient’s medical history. This offers a foundation for potential educational interventions aimed at improving emergency preparedness. Additionally, the study’s clear and interpretable results can inform curriculum development and future research in dental education.

However, the study has some limitations. The cross-sectional design limits the ability to infer causal relationships between variables, as it captures only a snapshot of participants’ knowledge and perceptions at a single point in time. Furthermore, while the sample size was adequate, it may not fully represent the broader population of dental students across different regions, as the study was limited to participants recruited from a single institution and the low response rate. The low response rate in this study may be due to timing, as it coincided with academic pressures like exams and clinical duties, which limited students’ availability to complete the questionnaire. Additionally, the self-administered online format may have led to lower engagement, as students may not have felt motivated to participate without more direct or incentivized encouragement. Additionally, despite the use of logistic regression, no significant impact was found for preparedness, suggesting that other unmeasured factors could be influencing students’ preparedness toward medical emergency management. Lastly, the reliance on self-reported data could introduce biases, as participants may overestimate or underestimate their actual preparedness or confidence in handling medical emergencies.

It is recommended that dental curricula are revised to include the incorporation of practical skills to clinical dental students throughout all years of clinical years. Clinical training can be enhanced by incorporating AI-driven virtual emergency simulations and even assigning students to shadow emergency response teams. As dental students enter their internship year, they can be engaged in short-term rotations in emergency departments, and participate in hospital “code blue” teams as observers or assistants.

## 5 Conclusion

It seems that there is a gap in the preparedness of dental students and interns to manage medical emergencies effectively. Enhanced training and education, particularly in managing more severe medical emergencies, is warranted to improve the overall emergency response in dental practices. Efforts should focus on increasing training and access to emergency drugs and equipment, along with regular BLS training. It is important to enhance emergency preparedness training for dental students, ensuring they are well-equipped to handle medical emergencies in clinical practice. Future research could consider the assessment of modern teaching strategies in topics of medical emergencies in enhancing skills and confidence of dental students.

## Data Availability

The raw data supporting the conclusions of this article will be made available by the authors, without undue reservation.
